# m^6^A/ m^1^A /m^5^C/m^7^G-related methylation modification patterns and immune characterization in prostate cancer

**DOI:** 10.3389/fphar.2022.1030766

**Published:** 2022-10-12

**Authors:** Xin Ye, Ruyi Wang, Xiaoqian Yu, Zili Wang, Haifeng Hu, Hanchao Zhang

**Affiliations:** ^1^ Department of Urology, Institute of Urology, West China Hospital of Sichuan University, Chengdu, China; ^2^ Department of Urology, The Affilated Hospital and Clinical Medical College of Chengdu University, Chengdu, China; ^3^ Molecular Medicine Research Center and National Clinical Research Center for Geriatrics, West China Hospital, and State Key Laboratory of Biotherapy, Sichuan University, Chengdu, China; ^4^ Medical College of Soochow University, Suzhou, China

**Keywords:** Prostate cancer, Methylation modification, Tumor microenvironment, Molecular subtype, Prognostic model

## Abstract

Methylation has a close relationship with immune reactions, metastasis, and cancer cell growth. Additionally, RNA methylation-related proteins have emerged as potential cancer therapeutic targets. The connection between the tumor microenvironment (TME) and methylation-related genes (MRGs) remains unclear. We explored the expression patterns of the MRGs in the genome and transcriptional fields of 796 prostate cancer (PCa) samples using two separate data sets. We identified a relationship between patient clinicopathological characteristics, prognosis, TME cell infiltrating qualities, and different MRG changes, as well as the identification of two distinct molecular groupings. Then, we formed an MRGs model to predict overall survival (OS), and we tested the accuracy of the model in patients with PCa. In addition, we developed a very accurate nomogram to improve the MRG model’s clinical applicability. The low-risk group had fewer tumor mutational burden (TMB), greater tumor immune dysfunction and exclusion (TIDE) ratings, fewer mutant genes, and better OS prospects. We discuss how MGRs may affect the prognosis, clinically important traits, TME, and immunotherapy responsiveness in PCa. In order to get a better understanding of MRGs in PCa, we could further explore the prognosis and create more effective immunotherapy regimens to open new avenues.

## Introduction

Prostate cancer (PCa) is the most frequent cancer diagnosis in men. Notably, PCa is the second most common neoplasm in senior men and the fifth leading cause of cancer-related mortality globally, accounting for 15% of all new tumor-related cases (Vietri et al., 2021). Most instances progress slowly and pose no danger to life. However, despite recent improvements, PCa still poses a serious medical challenge for the men affected. Therefore, finding novel prognostic indicators is essential for creating efficient treatment plans and enhancing PCa patients’ prognoses (Zhao et al., 2020).

Numerous biological processes, including cell differentiation, sex determination, stress response, and others, are known to be impacted by RNA methylation and its connected downstream signaling cascades (Menezo et al., 2020). RNA modification disorders have been linked to a wide range of cancers, including PCa (Haruehanroengra et al., 2020). As the third layer of epigenetics, more than 170 RNA modifications have been identified (Haruehanroengra et al., 2020). N6-methyladenosine (m^6^A), 5-methylcytosine (m^5^C), N1-methyladenosin (m^1^A), N7-methylguanosine (m^7^G) are post-transcriptional modifications, which are abundant in most eukaryotic mRNAs and involved in almost all stages of the RNA life cycle, including RNA transcription, translation and degradation. They are found in mRNA, lncRNA, and miRNA. Additionally, it is essential for the growth and development of numerous immune system illnesses, including cancers and a wide range of other human pathogenic activities (Dai et al., 2021). The evidence for RNA modification pathways being dysregulated in human malignancies is growing, and these pathways may provide excellent targets for cancer therapy (Barbieri and Kouzarides, 2020).

Fluctuations in RNA methylation in cancer are known as promising targets for developing useful diagnostic, prognostic and predictive biomarkers (Koch et al., 2018). It is also exciting to note that methylation has been connected to antitumor immunity in cancer immunotherapy (B. Yang et al., 2021). Besides necroptosis, methylation is also an important cellular response that controls the initiation, progression, and metastasis of cancer. Nevertheless, the role of some methylation regulators in the prognosis and possible molecular mechanisms of PCa is not well understood (B. Yang et al., 2021). Studying methylation landscapes can help predict the prognosis of PCa, according to Wen-Juan Li et al. (W. J. Li et al., 2021). A study identified 8 methylation-based biomarkers (cg04633600, cg05219445, cg05796128, cg10834205, cg16736826, cg23523811, cg23881697, cg24755931) which were useful for aggressively detecting PCa (Pu et al., 2021). To increase PCa cell survival and docetaxel resistance, SPOP mutations will upregulate the formation of stress particles (Shi et al., 2019). An invasive tumor is more likely to form in PCa with TP53 mutation (Maxwell et al., 2022). all of which are strongly methylation-deregulated and closely linked to prognosis. There are a few studies on the relationship between methylation and PCa, so we need to further study the fact that it plays a significant role in carcinogenesis and anticancer mechanisms.

Immunological checkpoint blocking, or immunotherapy (ICB, PD-1/L1 and CTLA-4), has shown astounding clinical success in a small minority of patients with long-term responses (Kalbasi et al., 2020). However, a large number of patients received little to no therapeutic benefit, which falls far short of satisfying a clinical need (M. Zhang et al., 2021a). It has only ever been assumed that the multi-step process of tumor formation alters the genetic and epigenetic makeup of tumor cells. But a large number of studies have shown us that the tumor microenvironment (TME) also has a significant role in the growth of the tumor (Vitale et al., 2019). Direct and indirect interactions between TME components can induce changes in biological behaviors such as immune tolerance (M. Zhang et al., 2021a). The MRG risk score for PCa was shown by Zhipeng Xu et al. colleagues to strongly correlate with immune infiltration (Xu et al., 2022). The decreased effectiveness of checkpoint inhibitors (CPIs) in advanced prostate cancer compared to other tumor types is likely largely due to an immunosuppressive tumor microenvironment (TME) and impaired cellular immunity (Bansal et al., 2021). The complexity and variability of the TME landscape should therefore be thoroughly parsed to identify various tumor immune phenotypes, which would also enhance the ability to predict and direct immunotherapeutic responsiveness (Hinshaw et al., 2019; Song et al., 2021). The discovery of very accurate biomarkers to gauge patients’ reactions to immunotherapy will aid in the search for novel therapeutic targets (Ehrlich, 2019).

We are now able to fully examine the transcriptome, metabolome, proteome, and genome in order to investigate the biomarkers and carcinogenesis framework for the therapy and prognosis of cancer when we explore the rapid advancement of science and the development of the Gene Expression Omnibus (GEO) and The Cancer Genome Atlas (TCGA) databases. We sought to determine MRG expression in PCa, prognostic importance, and putative regulatory axis. Our results may provide more information on the molecular processes and prognostic biomarkers of PCa.

## Materials and methods

### Data sources

From the TCGA (TCGA-PCa) and GEO (GSE65858 and GSE116918) databases, RNA-seq and clinicopathological data for PCa were retrieved (Supplementary Table S1). RNA-seq for PCa was converted to Transcripts Per Kilobase Million (TPM) values as previously mentioned and was taken into consideration to be equivalent to those for microarrays. After integrating two datasets (TCGA-PCa and GSE65858), batch effects were eliminated using the “Combat” method. The subsequent analyses included 796 PCa patients because we excluded data from people whose OS information was lacking or less than 30 days.

### Consensus clustering analysis of MRGs

These 84 MRGs are shown in Supplementary Table S2’s details. Using “ConsensusClusterPlus”, consensus unsupervised clustering analysis was utilized to divide patients into distinct molecular subgroups based on MRG expression. The following criteria were used to group these items: First, there was a fluid and progressive growth in the cumulative distribution function curve. Second, there was no tiny sample size in any group. Thirdly, although there was a drop in the inter-group correlation, the intra-group correlation rose. Gene set variation analysis (GSVA) was carried out to study MRG variation in biological processes.

### Correlation between clinical features and prognosis molecular subtypes

Age, gender, TNM stage, and clinical stage were some of the patient’s features. And to assess the two clusters identified by consensus clustering’s clinical value, we looked at the connections between molecular subtypes, clinical features, and prognosis. In addition, Kaplan-Meier curves, generated by the “survival” and “survminer” R programs, were used to compare OS among different subtypes.

### Relationship of molecular subtypes with TME

Additionally, the CIBERSORT algorithm was used to calculate the scores of 22 different human immune cell types for each PCa sample (Hao et al., 2019). We used single-sample Gene Set Enrichment Analysis (ssGSEA) to explore the levels of immune cell infiltration (Hwang et al., 2021). DEG identification and functional annotation of DEGs with the “limma” package in R, DEGs were discovered with a *p*-value of 0. 05 and |logFC| of 0. 585. We use the “clusterprofiler” package in R to perform functional enrichment analyses on the DEGs, allowing us to have a better analysis of the hidden functions of the methylation clusters in DEGs and discriminate between the enriched pathways and gene functions that go along with them.

### Construction of the prognostic risk model

We used unsupervised clustering to classify patients into different subtypes (gene cluster A and gene cluster B) for further study. All patients with PCA were randomly divided into training and testing groups with a ratio of 0.7:0.3 to establish a prognostic model. The DEGs were used in univariate Cox regression analysis in order to identify the DEGs associated with PCa’s OS. We employ the following procedures to calculate the risk score: Risk score is equal to (expi * coefi), where expi and coefi are the expression and risk coefficients of each gene, respectively. To lessen the possibility of over-fitting using prognostic DRGs, the LASSO Cox regression technique was temporarily used. In the two groups, the expression levels of genes connected to MRGs were examined. We divided patients into high-and low-risk score groups by the median of risk scores, and Kaplan-Meier analysis and receiver-operating characteristic (ROC) curves were used to assess the accuracy of risk scores. GSE116918 was applied as an external testing set to validate the model.

### Construction of a nomogram scoring system

We use the nomogram calibration plot to plot the forecast value between 3-, 5-, and 8-year survival events and virtual observations. A variable in a nomogram scoring system that combines risk scores and clinical characteristics has a score, and the total score is the sum of all the individual scores (Iasonos et al., 2008).

### Mutation, immunotherapy response and drug susceptibility analysis

It is investigated how the genes in the model relate to the 22 immune cells. The ESTIMATE algorithm was applied to assess the immune and stromal scores of each sample. The TCGA database generates mutation annotation formats to identify somatic mutations in various PCa sample groups. We determine the tumor burden mutation (TBM) score for each PCa patient across the two categories. We investigated the associations between tumor immune dysfunction and exclusion (TIDE) and different groups. We created the half-maximal semi-inhibitory concentration (IC50) values of a pRRophetic package of anti-tumor medications for PCa in order to examine the difference in the treatment impact of commonly used anti-tumor agents between the two groups.

## Results

### Genetic and transcriptional alterations of MRGs in PCa

**FIGURE 1:**
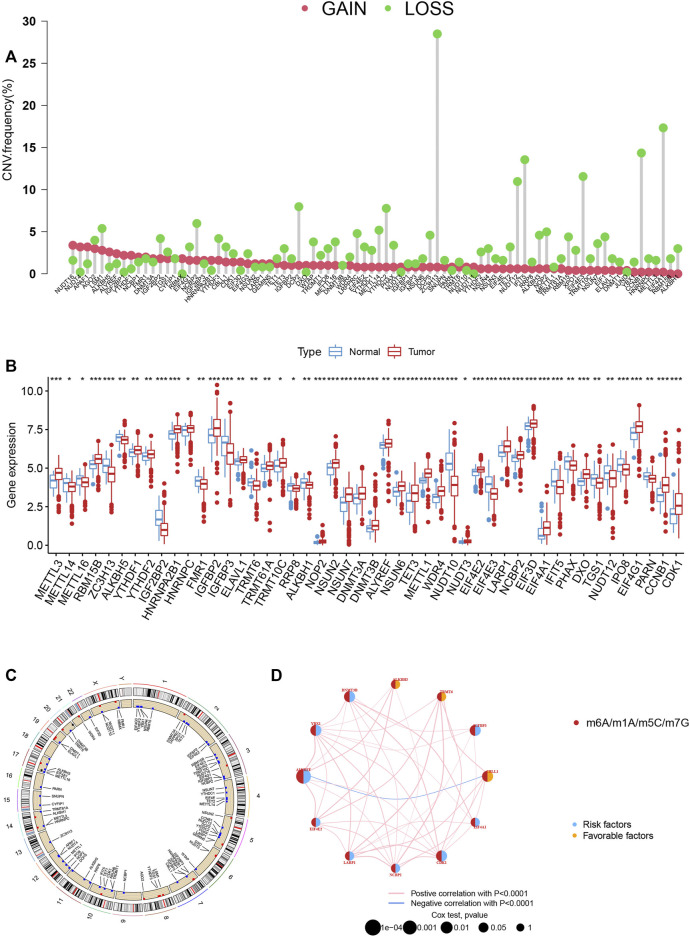
**(A)** The CNV of 84 MRGs. **(B) **Expression distributions of differentially expressed MRGs between normal and PCa tissues. **(C)** The positions of the CNV alterations on their respective chromosomes for these MRGs. **(D)** The overall group of MRG interactions, regulatory factor connectivity and value of prognosis in PCa patients was identified in the network.

According to the analysis, we could see significant differences in the potential function of MRGs in PCa carcinogenesis with the expression levels and genetic landscape of MRGs between PCa and control samples. In this investigation, 84 MRGs were examined (Supplementary Table S2). We then looked into somatic copy number variation in the 84 MRGs and discovered that there were a number of common copy number alterations, including increases in general copy number variation (CNV) in NUDT16, NUDT4, APAF1, AGO2, LSM1, and ALKBH5, and decreases in CNV in ZC3H13, ELF4A1, CCNB1, IFIT5, ELF4E3, and NUDT12 (Figure 1A). MRGs with CNV loss were expressed at lower levels, such as ZC3H13, IFIT5, ELF4E3 and NUDT12 in PCa samples, when compared to those in normal PCa samples (Figure 1B), hinting that the mRNA expression of MRGs might be regulated by CNV. Figure 1C shows the locations of CNV alterations on their respective chromosomes in MRGs. DNA methylation factors could modulate gene expression (Nishiyama and Nakaanishi., 2021).

### Identification of methylation-related subtypes

**FIGURE 2:**
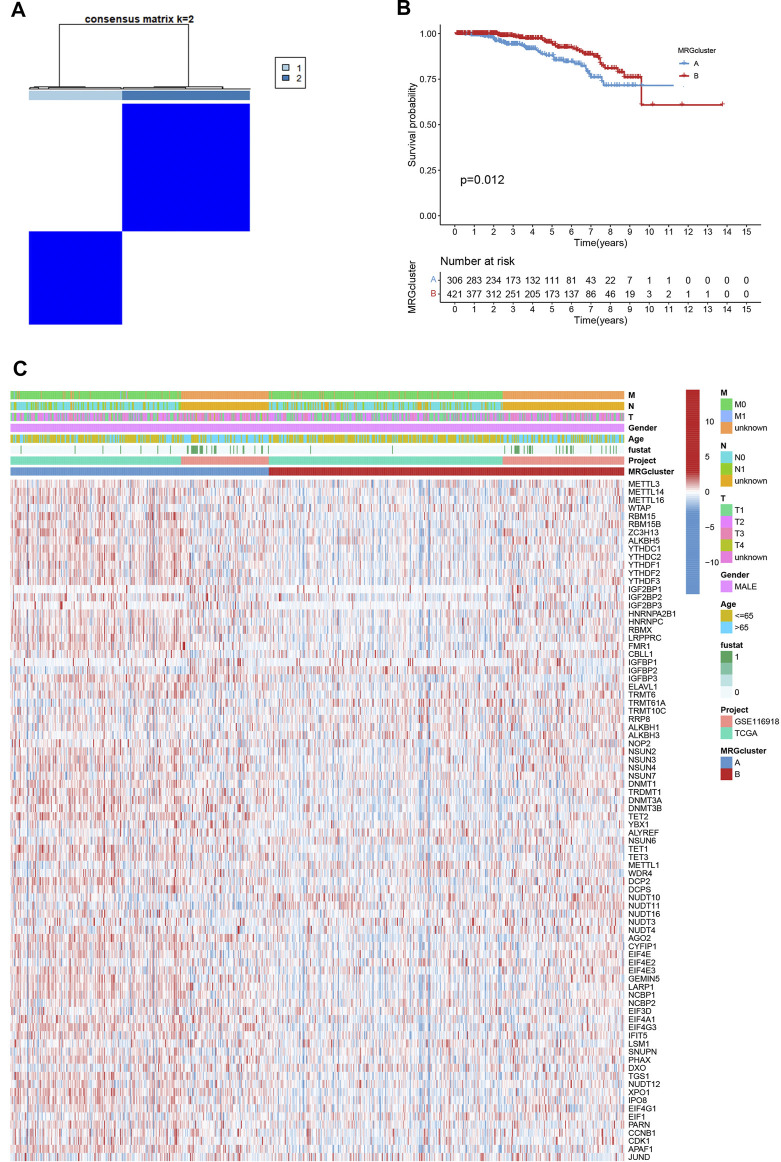
**(A)** Consensus matrix heatmap defining two MRG clusters (*k* = 2). **(B)** Kaplan-Meier curves indicated a shorter OS in patients with MRG cluster A than in patients with MRG cluster **(B) (C)** Differences in clinical features and MRG expression levels between the two MRG subtypes.

We picked 796 patients (TCGA and GSE116918) to explore the expression pattern of MRG involved in tumorigenesis for further analysis. The 12 prognostic MRGs were recognized by univariate Cox analysis. The prognostic MRG interactions, regulatory factor connectivity and value of methylation in PCa patients were identified in the methylation network (Figure 1D). Based on the 84 MRGs’ expression profiles, we used a consensus clustering approach to classify the PCa patients. We classified the entire cohort as the best choice for MRG cluster A and B based on *k* = 2 (Figure 2A and Supplementary Figure S1). Patients in MRG Group B had a better OS, as hinted by the Kaplan-Meier curves (*p* = 0. 012; Figure 2B). Furthermore, we demonstrate that MRG expression and clinical pathology characteristics are significantly different (Figure 2C).

### Evaluation of TME

**FIGURE 3:**
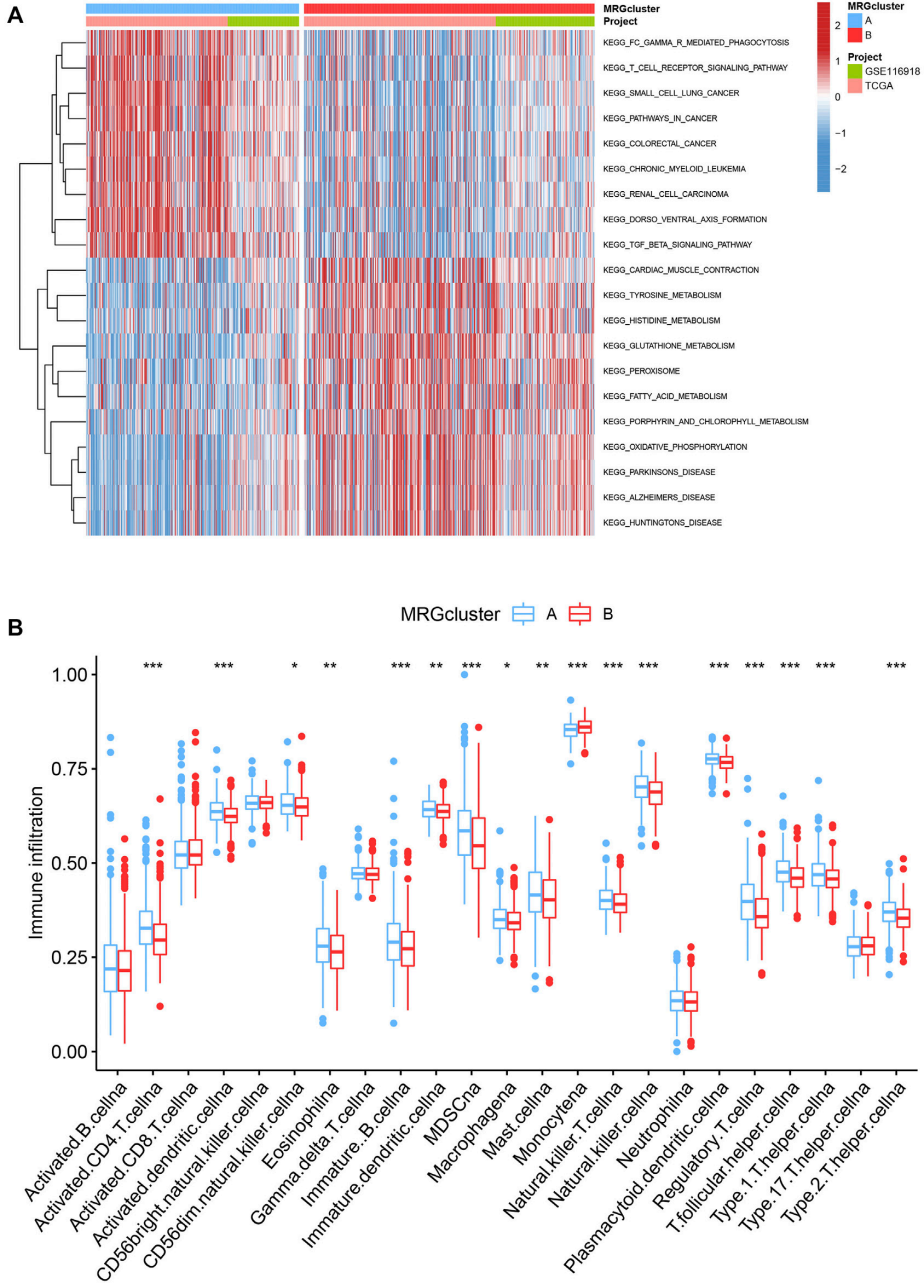
**(A)** Heatmap of GSVA enrichment analysis results. **(B)** Significant differences occurred among the two subtypes in the infiltration of some immune cells.

GSVA enrichment analysis showed that MRG cluster B and MRG cluster A were significantly different. One was in fc gamma r mediated phagocytosis, T cell receptor signaling pathway, small cell lung cancer, and pathways in cancer, while another was in huntingtons disease, alzheimers disease, parkinsons disease, and oxidsative phosphorylation (Figure 3A). We examine the relationships between the 22 human immune cell subsets and the two subtypes of each PCa sample by using the CIBERSORT method. There were important variations between the two subtypes in terms of the invasion of certain immune cells. (Figure 3B).

### Classification of gene clusters

**FIGURE 4:**
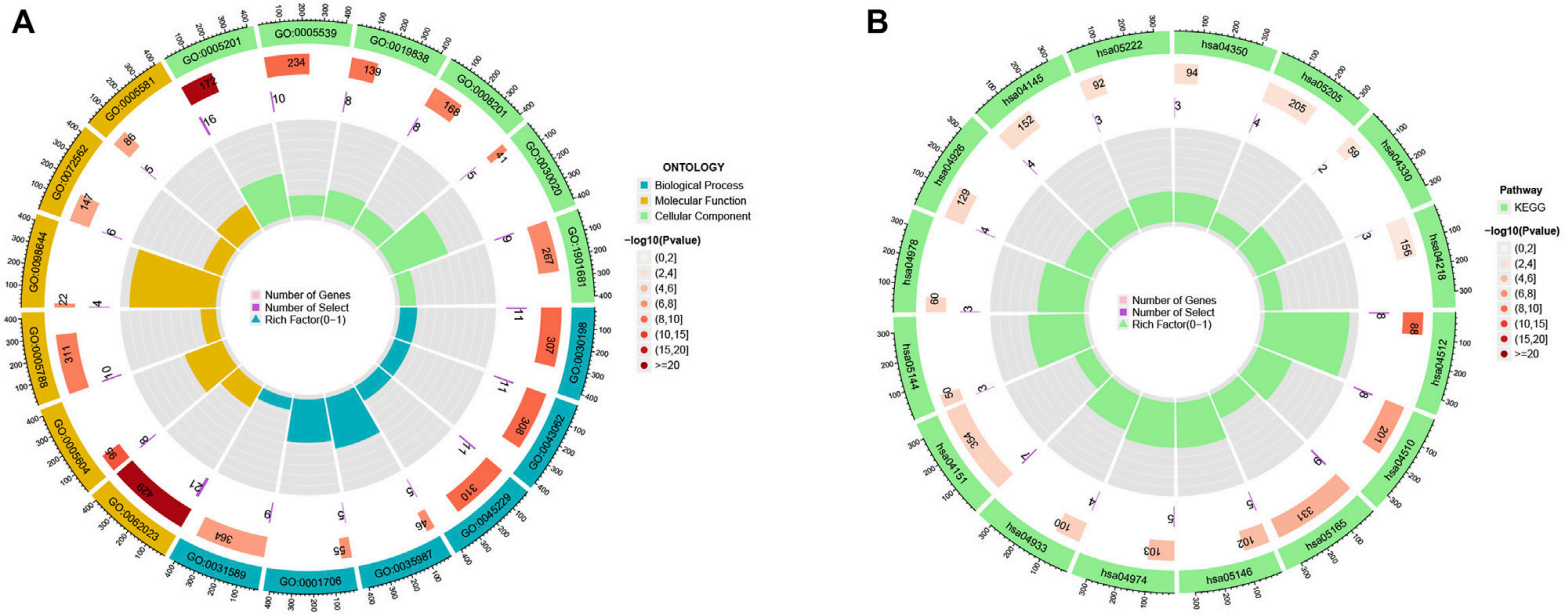
**(A–B)** GO and KEGG enrichment analyses.

**FIGURE 5:**
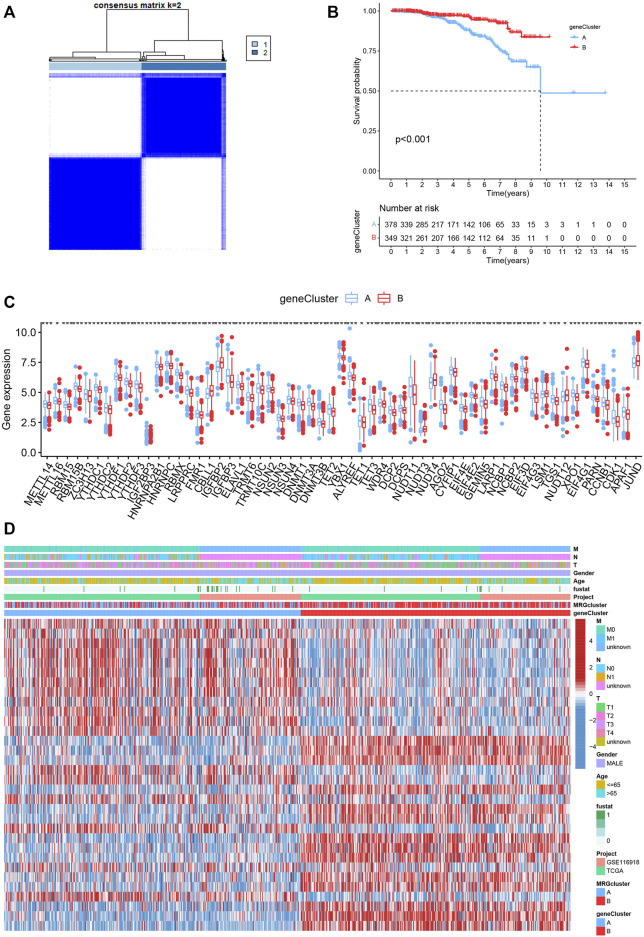
**(A)** Consensus matrix heatmap defining two MRG clusters (*k* = 2). **(B)** Kaplan-Meier curves indicated that patients with gene cluster B had higher OS. **(C) **The expression levels of MRGs in the two gene subtypes. **(D)** Differences in clinical features and MRG expression levels between the gene subtypes.

To investigate the underlying biological behaviour of each focal flash pattern, the R package “limma” was used to recognize 74 DEGs linked to MRG subtypes. These were then subjected to functional enrichment analysis (Figures 4A,B). These DEGs were widely distributed in biological processes and were associated with immunity (Figure 4A and Supplementary Table S3). KEGG analysis revealed an enrichment of immunological and cancer-related pathways, demonstrating the significance of methylation in the immune control of the TME (Figure 4B and Supplementary Table S4). By using univariate Cox regression analysis, 32 prognostic DEGs related to OS time were chosen from 74 DEGs (*p* < 0.05; Supplementary Table S5). In order to validate these regulatory mechanisms, consensus clustering techniques were utilized to share patients into two gene categories based on prognostic genes (Figure 5A and Supplementary Figure S2). According to Kaplan-Meier curves (*p* < 0. 001; Figure 5B), patients with gene cluster B had the highest OS, which is obviously better than that of cluster A. The two gene subtypes’ MRG expression showed significant variety, which was in line with our predictions (Figure 5C.) Additionally, a comparison of the clinicopathological characteristics of several gene subtypes revealed a substantial difference between clinical aspects and gene expression (Figure 5D).

### Construction and validation of the prognostic risk model

**FIGURE 6:**
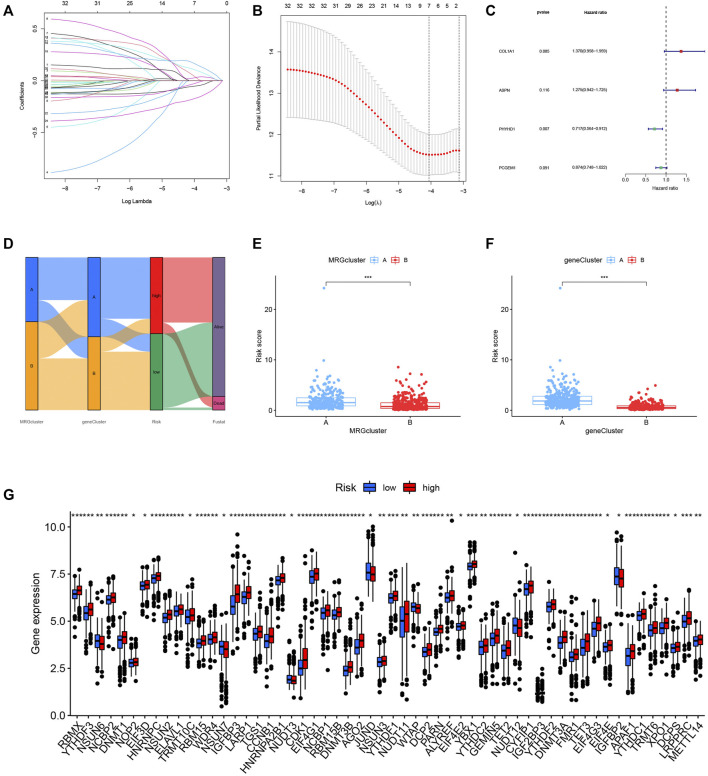
**(A–C)** The model was constructed by LASSO and multivariate Cox regression analysis. **(D)** The relationship between MRG cluster, gene cluster, risk groups, and survival status was visualized in a Sankey diagram. **(E–F)** The distribution of risk scores for the two subgroups. **(G)** The expression of ICIs-related genes was significantly different between groups.

We randomly grouped the patients into training and testing groups in a ratio of 0. 7: 0. 3 (“caret package” in R). To further narrow down the best prognostic signature, the prognostic DEGs were run through LASSO and multivariate Cox analysis (Figures 6A–C). The risk model was built using the following steps: risk score = (0.315* COL1A1) + (0.243* ASPN) + (-0.333* PHYHD1) + (-0.134* PCGEM1). A Sankey diagram was used to illustrate the relationship between the MRG cluster, gene cluster, risk groups, and survival status (Figure 6D). The risk score distributions for the two categories are shown in Figures 6E,F. We found that the expression of MRGs varied considerably between groups (Figure 6G).

**FIGURE 7:**
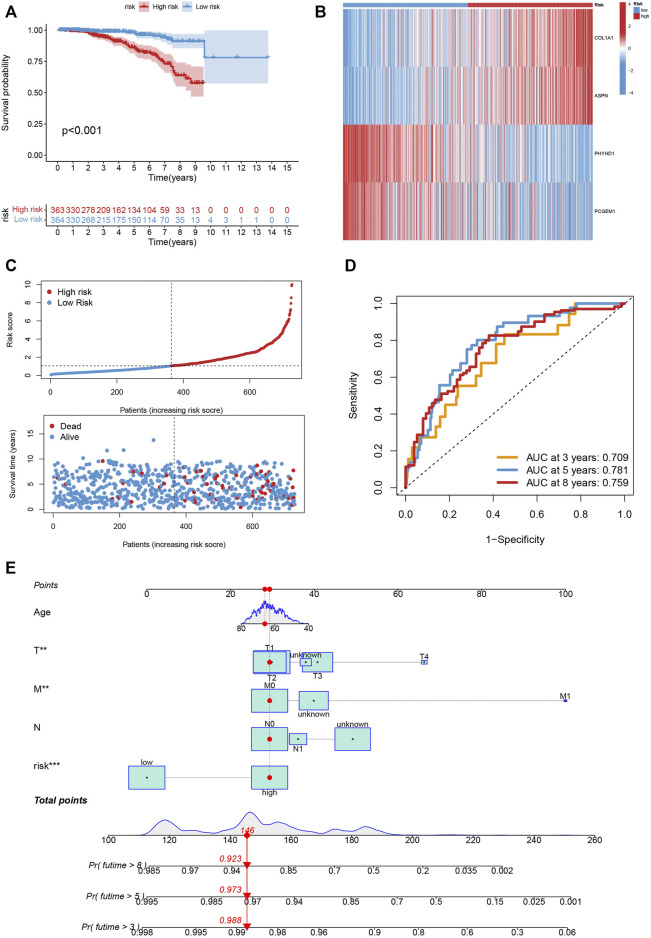
**(A–C)** The Kaplan-Meier analysis, expression profiles, pattern of survival status, and the distribution of risk scores in the entire cohort. **(D)** The ROC curves for the 3, 5, and 8-year AUC values in the entire cohort. **(E)** The nomogram containing the model and clinical features was reliable and sensitive for predicting survival in patients with PCa.

The Kaplan-Meier analysis, expression profiles, pattern of survival status, and distribution of risk scores are shared in Figures 7A–C, which hints that patients in the low-risk category will live longer. The model’s high sensitivity and specificity for predicting survival were demonstrated by the ROC curves, and the overall set’s 8-year AUC value was 0. 759 (Figure 7D). In Supplementary Figures S3–S5, which provide the above analysis for the training, testing and external testing sets, the model’s dependability is shown. Figure 7E was the nomogram that included the model and clinical characteristics.

### Evaluation of TME

**FIGURE 8:**
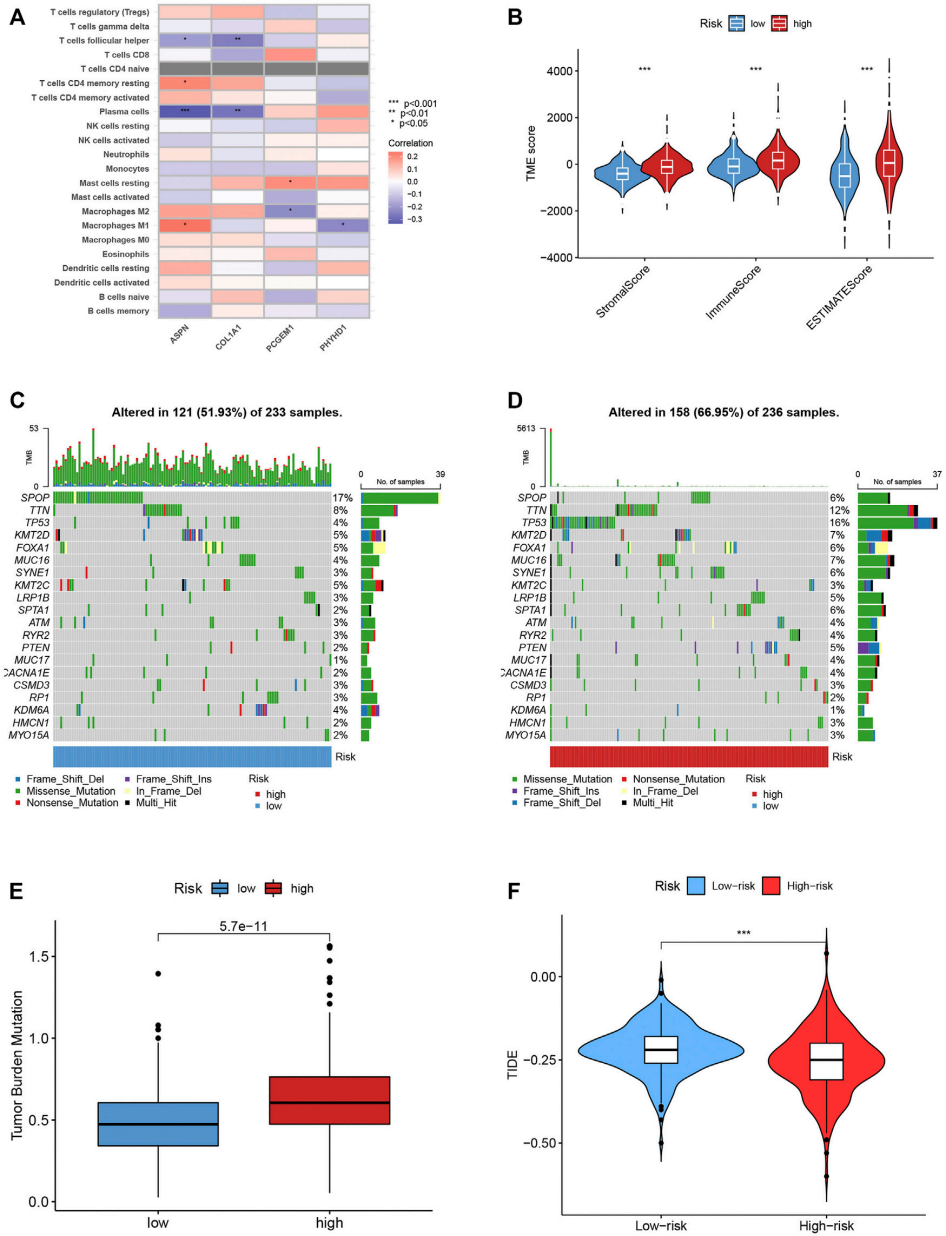
**(A)** The connection between the number of immune cells and the 4 genes in the model. **(B)** The high-risk scores were linked to a low stromal score, and the low-risk scores was highly correlated with a high immune score. **(C–D)** In the high- and low-risk groups, the top 10 mutant genes were SPOP, TTN, TP53, KMT2D, FOXA1, MUC16, SYNE1, KMT2C, LRP1B and SPTA1. **(E)** TBM score between different groups. **(F)** TIDE scores were lower in the high-risk score group, suggesting that the high-risk score group was more responsive to immunotherapy.

We also looked at the relationship between the number of immune cells and the four genes in the proposed model, and found that the majority of immune cells are obviously related to the four genes (Figure 8A). The low-risk score group was strongly correlated with a low immunological score, while the high-risk score group was linked to a high stromal score (Figure 8B).

### Mutation, immunotherapy response and drug susceptibility analysis

We examined how the TCGA-PCa cohort’s various risk score groups differed in the somatic mutation distribution. The top 10 mutant genes in the high- and low-risk categories were SPOP, TTN, TP53, KMT2D, FOXA1, MUC16, SYNE1, KMT2C, LRP1B, and SPTA1 (Figures 8C,D). Patients in the low-risk score group had considerably higher frequencies of SPOP mutations compared to those in the high-risk score group. Further, high TBM was connected with poor OS (*p* < 0.001; Figure 8E). The high-risk score group had lower TIDE scores, indicating that they might have responded better to immunotherapy (Figure 8F). Furthermore, by examining the IC50 of regularly used anticancer medicines, we found a significant difference between the two patient groups’ susceptibility to the treatments. (Supplementary Figure S6).

## Discussion

*In vitro* and *in vivo* tumor growth, invasion, migration, and the epithelial-mesenchymal transition of cancer cells are all influenced by dynamic RNA methylation and modification events, such as m^6^A, m^1^A, m^5^C and m^7^G (X. Y. Li et al., 2022; Traube et al., 2017). In addition to playing essential roles in various cancers and anticancer effects, modification events can also be used as prognostic indicators (Mahmoud and Ali, 2019). There are still several unanswered questions regarding the overall effect and the features of TME penetration adjusted by the effects of numerous MRGs (M. Li et al., 2021).

We identified two distinct molecular subgroups using 84 MRGs. And patients with subtype B had a better OS. The features of the TME varied obviously across the two subtypes. Variations in mRNA transcriptomes between different methylation subtypes were strongly linked with biological pathways involved in MRG and the immune system (Gu et al., 2021; X. Y. Li et al., 2022). We determined two gene subtypes relied on the DEGs between the two methylation subtypes. According to the data, MRGs may be utilized to predict PCa’s clinical prognosis and responsiveness to treatment (Zhang et al., 2020). As a result, we discovered and validated the accurate prognostic MRG-score. Higher and lower MRG-scores were seen in immune activation- and inhibition-driven PCa patterns, respectively. Finally, we combined the risk score and tumor stage to produce a quantitative nomogram, which dramatically improved performance and made it simpler to utilize the risk score (Jeong et al., 2020).

A growing body of research has established that MRG alteration played a significant role in the post-transcriptional modification of gene expression, which was strongly associated with tumor formation, maintenance, progression, and prognosis, thanks to advancements in detecting technology. As reported, high TET3 expression (m^5^C-related gene) was related to poor prognosis of PCa (Yu et al., 2022). According to certain research, m^6^A alteration significantly influences the stability of mRNA, which in turn contributes to PCa development (Du C et al., 2020). PCa bone metastases were related to high m^6^A levels of NEAT1-1, and m^6^A levels of NEAT1-1 were a reliable indicator of ultimate death (Wen et al., 2020). In recent years, m^7^G has been thought to be actively implicated in cancer-related translation problems. The m^7^G-score has been shown to be an independent measure of BCR-free survival in patients with PCa (Xin et al., 2022). Additionally, recent research has shown that RNA modification regulators may serve as biomarkers for cancer diagnosis and prognosis surveillance (Haruehanroengra et al., 2020). Nevertheless, a thorough examination of the prognostic significance and functional annotation of MRGs regulators in PCa is still lacking.

PCa patients’ prognoses are poor. There were significant differences between patient subgroups in terms of TME, immunological checkpoints, CSC index, prognosis, mutation, and therapy susceptibility after standard therapy because of high levels of checkpoints, lymphocytes that infiltrate tumors, and tumor neoantigens (D. Li et al., 2022). Despite recent developments in immunotherapy, patients with PCa still experience heterogeneity in their results, underlining the important role of TME in the growth and development of PCa tumors (Yu et al., 2022). Immune cells, including granulocytes, lymphocytes, and macrophages, are important biological components of TME. These cells participate in a variety of immunological responses and behaviors, such as the inflammatory response that tumors trigger to help them survive (Schmitt and Greten., 2021). Additional data points to the TME having a significant impact on cancer development, progression, and therapeutic resistance (Cao et al., 2021; Martínez-Reyes and Chandel., 2021). Immune inhibition-driven methylation (subtype A) was associated with a higher risk score, whereas immune activation-driven methylation (subtype B) was related to a lower risk score. We discovered that the relative richness of 22 immune cells as well as the two molecular subtypes’ differences in risk scores and TME traits were significantly different.

Various kinds of T cells are crucial components of the immune defense against PCa (K. Yang and Kaliies., 2021). Higher densities indicated a positive prognosis as tumor-infiltrating T cell densities in PCa samples were higher than those in normal tissues (Yu et al., 2022). The enhanced infiltration of activated memory CD4^+^ and CD8^+^ T cells as well as gamma delta T cells was seen in the subtype B and low risk score groups, indicating that they favourably contribute to the progression of PCa. A worse prognosis was associated with Treg infiltration, which blocks the immune system’s anti-cancer response (Oh and Fong., 2021). This is in line with our observation that patients in the high-risk group and those with subtype B had more Tregs in the TME than those in the low-risk group. Recently, it was shown that B cells aid in the immune response (Fridman et al., 2022; Zhang et al., 2022).

Petitprez et al. believed that in soft-tissue sarcomas, the response to PD-1 inhibition was positively linked with B cell enrichment (Petitprez et al., 2020). Patients who responded to immune checkpoint blockade showed considerably higher levels of the B cell-related genes than those who did not, according to Helmink et al. (Fridman et al., 2022). Additionally, in PCa, tumor-infiltrating B lymphocytes were linked to a good prognosis (Horii et al., 2021). Patients with significant B cell infiltration in their metastatic PCa had prolonged overall survival and a significantly lower risk of the disease coming back (Engelhard et al., 2021). The results of this study demonstrated that B cells are not only incidental contributors to anti-cancer immunotherapy; rather, they present a novel immunotherapy target and may be a potent cancer-fighting tool. In our study, we found subtype B had considerably fewer naive B cells and higher MRG-score, which were associated with poorer overall survival (Franchina et al., 2018).

In this study, the expression levels of a part of immune cells were found to be obviously different in the risk model of MRGs. The stromal score, CD4 memory resting T cells, CD4 memory activated T cells, follicular helper T cells, M0 macrophages, M1 macrophages, and resting mast cells were linked with the risk score. This implies that PCa immune cell infiltration is related to the risk model created using MRGs (He et al., 2022). Our study shows that differentially expressed ASPN,COL1A1, PCGEM1 and PHYHD1 was associated with immune infiltration. The high-risk score group was related to a high stromal score, and the low-risk score group was closely associated with a high immune score. Pu Zhang et al. showed that while ASPN is overexpressed in PCa, a bad prognosis is predicted by excessively high ASPN expression and low expression of other genes, ASPN is independently associated with overall survival (OS) of patients (P. Zhang et al., 2021b). High expression of COL1A1 can predict the prognosis of cancer and is a reliable biomarker and therapeutic target (Ma et al., 2019; Geng et al., 2021). And many studies have shown that the high expression of PCGEM1 and PHYHD1 can promote the value-added migration and invasion of cancer, affecting prognosis (Jiang et al., 2019; Zhang et al., 2019; Liu et al., 2022). Our study identified the involvement of MRGs and constructed a risk model for PCa. However, this must be confirmed using additional clinical PCa tissue samples and cell experiments. MRGs are generally involved in the occurrence and development of PCa. An independent risk factor for a bad prognosis in PCa patients and a high-risk score is related to patient outcome (Chong et al., 2021). The risk score is associated with PCa stromal score and levels of CD4 memory resting T cells, M0 macrophages, M1 macrophages, resting mast cells, CD4 memory activated T cells, and follicular helper T cells (Xu et al., 2021).

The investigation suffered from a variety of flaws. First and foremost, the samples applied in our investigation were collected retrospectively, all the outcomes were obtained using only data from public databases, and validation in a separate clinical patient cohort is still lacking despite the use of external datasets for validation. Next, surgery, neoadjuvant chemotherapy, and chemoradiotherapy, which may have affected how well the immune response and methylation condition performed.

## Conclusion

Here, we disclosure the roles of MRGs modification patterns in the PCa and TME diversity, clinicopathological characteristics and a wide range of prognostic regulatory mechanisms. Next, the therapeutic obligations of MRGs in immunotherapy and commonly used antineoplastic drugs are explained by us. These discoveries emphasize the key clinical significance of MRGs, which offer a new view into the field of PCa research and promote the understanding of TME and immunotherapy in the future.

## Data Availability

The original contributions presented in the study are included in the article/Supplementary Material, further inquiries can be directed to the corresponding author.

## Supplementary material

SUPPLEMENTARY TABLE S1 The details of clinical characteristics.

SUPPLEMENTARY TABLE S2 The list of MRGs.

SUPPLEMENTARY TABLE S3 The details of GO analysis.

SUPPLEMENTARY TABLE S4 The details of KEGG analysis.

SUPPLEMENTARY TABLE S5 32 prognostic DEGs by univariate Cox analysis.
